# Antimicrobial Susceptibility Profiles among *Pseudomonas aeruginosa* Isolated from Professional SCUBA Divers with Otitis Externa, Swimming Pools and the Ocean at a Diving Operation in South Africa

**DOI:** 10.3390/pathogens11010091

**Published:** 2022-01-13

**Authors:** Kevin Maclean, Fernande Olpa J Pankendem Njamo, Mahloro Hope Serepa-Dlamini, Kulsum Kondiah, Ezekiel Green

**Affiliations:** Department of Biotechnology and Food Technology, Faculty of Science, Doornfontein Campus, University of Johannesburg, Doornfontein 2028, Johannesburg P.O. Box 17011, South Africa; kevinm@uj.ac.za (K.M.); 218026023@student.uj.ac.za (F.O.J.P.N.); hopes@uj.ac.za (M.H.S.-D.); egreen@uj.ac.za (E.G.)

**Keywords:** antimicrobial resistance, beta lactamase, divers, otitis externa, swimmer’s ear

## Abstract

SCUBA divers are predisposed to otitis externa caused by *Pseudomonas aeruginosa,* which is becoming increasingly multi-drug resistant (MDR). The present work assessed the antibiotic resistance profiles of *P. aeruginosa* obtained from SCUBA divers and their environment in Sodwana Bay, South Africa. Bacterial isolates from a total of 137 random water and ear swab samples were identified using biochemical and molecular methods. *P. aeruginosa* strains were further evaluated for antibiotic susceptibility using the Kirby–Bauer assay. Double disk synergy test (DDST) to confirm metallo-β-lactamase (MBL) production and PCR amplification of specific antibiotic resistance genes was performed. All (100%) 22 *P. aeruginosa* isolates recovered were resistant to 6 of the β-lactams tested including imipenem but exhibited susceptibility to trimethoprim–sulfamethoxazole. MBL production was observed in 77% of isolates while the most prevalent extended-spectrum β-lactamase (ESBL) genes present included *bla_AmpC_* (86.9%) followed by *bla_TEM_* (82.6%). Sulfonamide resistance was largely encoded by *sul1* (63.6%) and *sul2* (77.3%) genes with a high abundance of class 1 integrons (77.3%) of which 18.2% carried both *Intl1* and *Intl2*. *P. aeruginosa* found in Sodwana Bay exhibits multi-drug resistance (MDRce) to several pharmaceutically important drugs with the potential to transfer antibiotic resistance to other bacteria if the judicious use of antibiotics for their treatment is not practiced.

## 1. Introduction

Swimming and other water activities are popular social recreational activities. Swimming pool water is decontaminated to remove harmful bacteria. Nevertheless, certain persistent bacteria survive in water [1] and are able to infect people who use that water for recreational purposes. Self-contained-underwater-breathing-apparatus (SCUBA) diving is a very common aquatic attraction. Approximately 15 million SCUBA dives are conducted on an annual basis worldwide [2]. SCUBA diving has become part of tourism in South Africa, especially in Sodwana Bay in KwaZulu Natal, South Africa. A previous study conducted between July 2011 and July 2012 indicated that a total of 59,553 dives were conducted by 15,789 divers, which resulted in a total value of USD 404,876.53 in Sodwana Bay [3]. Swimming pool water can be contaminated with micro-organisms from the surroundings including bathers [4], making recreational water a source of infectious diseases such as acute gastrointestinal, cutaneous, and respiratory illnesses caused by several micro-organisms [4].

Members of the genus *Pseudomonas* are commonly found in the environment, including in 10% of human stools, and on the skin of some healthy humans [5]. *Pseudomonas aeruginosa* has been implicated in infections of the urinary tract, pneumonia, wound infections, and otitis externa throughout the world [6,7,8]. Otitis externa (OE, swimmer’s ear) is an infection of the outer ear attributable to bacteria resulting in irritation of the mucosa layer. The infection might be aggressive as it can rapidly spread outside the external auditory canal, termed malignant otitis externa. Malignant Otitis Externa is an expansive illness with possible involvement of the base of the skull as well as a threat to life [9]. *P. aeruginosa* has become the most frequently identified pathogen in the development of malignant otitis externa [10]. However, drug resistance is becoming a serious problem in the treatment of *P. aeruginosa.*

The development of increased resistance in hospital environments [3], in particular, multidrug-resistance (MDRce), exacerbates anti-pseudomonas chemotherapy. It is clear from literature that *P. aeruginosa* has developed resistance against large families of antimicrobial agents, such as the β-lactams [11,12]. The increasing frequency of drug resistance and MDR *P. aeruginosa* constitute a risk in antibiotic therapy [11]. The eight types of antibiotics used to treat *P. aeruginosa* infections include: Aminoglycosides (Gentamicin, Tobramycin, Amikacin), Carbapenems (Imipenem, Meropenem), Cephalosporins (Ceftazidime, Cefepime), Fluoroquinolones (Ciprofloxacin, Levofloxacin), Penicillins with β-lactamase inhibitors (BLI) (Ticarcillin and Piperacillin in combination with Clavulanic acid or Tazobactam) [12], Monobactams (Aztreonam), Fosfomycin, and Polymyxins (Colistin, Polymyxin B). The WHO listed carbapenem resistant *P. aeruginosa* as a priority pathogen [13]. In the past decade, *P. aeruginosa* has been shown to be increasing in resistance due to the presence of various classes of β-lactamases, namely, A, B, and D [14], which include both extended-spectrum β-lactamases (ESBLs) and metallo-β-lactamases (MBLs). Treatment of MDR *P. aeruginosa,* therefore, relies on combination therapy frequently combining a β-lactam with an aminoglycoside, for example, ceftolozane and tazobactam or alternatively imipenem, amikacin, and cefepime among others [15]. The aim of this study was to determine the susceptibility profiles of *P. aeruginosa* isolated from professional SCUBA divers, ocean, and swimming pool water from Sodwana Bay, KwaZulu Natal, South Africa, to different classes of antibiotics.

## 2. Results and Discussion

### 2.1. Identification of P. aeruginosa

A total of 22 isolates were identified as *P. aeruginosa* and 1 as *P. putida.* From the positively identified *P. aeruginosa* isolates, eight (36.4%) were isolated from divers with OE (OE), one of which was a tourist (T_w_OE) and two (9.1%) isolates were obtained from divers after their first pool training (APT). A single (4.5%) isolate was obtained from pool water (PW) before the first pool training while two (9.1%) were isolated from PW after the first pool training. An additional two (9.1%) *P. aeruginosa* were isolated from the PW 24 h after chlorination. Three isolates (13.6%) of *P. aeruginosa* were found in surface samples of ocean water (OSW) and a further four (18.2%) isolates were identified in ocean water 15 to 20 m deep (OBW). These results correlate well to some of the natural habitats in which *P. aeruginosa* thrives, i.e., water, and the microbiota found on human skin.

16S rDNA sequencing revealed that all the isolates phenotypically identified as *P. aeruginosa* shared a 98% to 100% similarity to *P. aeruginosa* strains in GenBank. Being ubiquitous [14], it was not a surprise that *P. aeruginosa* was isolated from the different sources sampled. The variable genetic makeup of *P. aeruginosa* makes it possible to colonize many habitats including people. For example, *P. aeruginosa* has been reported as being one of the most prevalent antibiotic resistant pathogens responsible for a wide range of hospital acquired infections in critically ill patients within the intensive care unit [15]. It rarely affects healthy people, yet leads to elevated morbidity and deaths in immunodeficient people [16]. In this study, a total of 45.5% of the isolates emanated from the divers; a majority of which, as expected, originated from divers that had presented with OE.

### 2.2. Antimicrobial Sensitivity Profiling

Disk diffusion was used to determine the sensitivity of 22 *P. aeruginosa* isolates to different classes of antibiotics that included the β-lactams, aminoglycosides, quinolones, glycopeptides, and sulfonamides. Table 1 shows the antimicrobial susceptibility profiles of *P. aeruginosa* where resistance to all antibiotics tested was evident except for Bactrim (trimethoprim-sulfamethoxazole) to which all isolates were susceptible. All twenty-two (100%) *P. aeruginosa* isolates were resistant to six of the ten β-lactams tested including imipenem. Only twelve (54.5%) isolates exhibited resistance to meropenem. High resistance to β-lactams was also reported in patient samples collected from a private hospital in Durban, South Africa [17]. Of seventeen *P. aeruginosa* isolates, 94% were resistant to aztreonam, 82% to meropenem and 76% to piperacillin–tazobactam. In addition, 82% of these isolates also showed resistance to ciprofloxacin. In addition to the presence of ESBLs and MBLs, resistance was attributed to other mechanisms such as impermeability and/or efflux pumps. A review by Ekwanzala et al. [18] goes on to report that in KwaZulu Natal, the distribution frequency of the various classes of AMR includes 9% each for tetracyclines, sulfonamides, and carbapenems; 18% for ESBLs; and 55% for MDR organisms. Both imipenem and meropenem are β-lactam antibiotics that play a key role in the medical treatment of *P. aeruginosa* infections due to their demonstrated effectiveness and protection. The resistance of *P. aeruginosa* to these β-lactams could be mediated by one of two chromosomal mechanisms. The OprD porine is said to promote the internalization of imipenem and some meropenem, but not the assimilation of all β-lactam. Changes to the composition of OprD and/or a decrease in expression contribute to a decline in imipenem sensitivity [19]. Moreover, OprD changes are related to excess expression of the efflux mechanisms increasing meropenem resistance, and thus such bacteria have a high degree of tolerance to these carbapenems and perhaps other antibiotic types, such as quinolones and aminoglycosides [20]. Carbapenem resistant *P. aeruginosa* is, thus, usually insensitive to most antibacterial agent types, including polymyxins and tigecycline. Regrettably, elevated levels of toxicity and under optimal bioavailability restrict the use of polymyxins and tigecycline. New treatment strategies such as ceftolozane–tazobactam are, thus, becoming important for the treatment of carbapenem resistant *P. aeruginosa*.

Interestingly, all 22 isolates were susceptible to Bactrim although *P. aeruginosa* is known to use drug efflux to resist trimethoprim–sulfamethoxazole. Studies in livestock [21] or their products [22] have recorded some susceptibility of *P. aeruginosa* isolates to trimethoprim–sulfamethoxazole. Susceptibility to trimethoprim–sulfamethoxazole was also reported in *P. aeruginosa* isolated from patients with cystic fibrosis [23]. While majority of the susceptible isolates emanated from patients with cystic fibrosis (an increase of 34 to 68% from 1999 to 2011), a number were also present in non-cystic fibrosis patients. The susceptibility to trimethoprim–sulfamethoxazole was attributed to mutations in *mex* gene determinants, and in *mutL* and *mutS*. A similar characterization of critical mutations in *mexR*, *mexAB-oprM*, *mexZ,* and *mut* genes would confirm the sensitivity of the *P. aeruginosa* isolates in the present study to Bactrim. 

### 2.3. Detection of ESBL and MBL Production

Positive DDST results for MBL production were observed from 17 (77%) of the 22 isolates tested. Infections resulting from MDR *P*. *aeruginosa* are usually treated with carbapenems, but the increase in MBL prevalence has made its treatment with this class of last line antibiotics increasingly challenging. The added MDRce, evident even in the isolates from this study, further exacerbates the challenges associated with its treatment. MBL producing *Pseudomonas* species continue to be an important organism causing life-threatening infections, and therefore the use of DDST for routine screening of *P. aeruginosa* associated with OE in SCUBA divers at dive sites is recommended. The findings from this study indicate that in Sodwana Bay, the treatment of OE causing *P. aeruginosa* using the current prescribed antibiotics may not be effective in clearing the infections. This may be the case at other dive sites both nationally and globally. Sending samples to the laboratory for PCR testing to confirm MDRce in the causative agents would incur higher costs and increase diagnosis time, which would deter recreational SCUBA divers from seeking immediate medical assistance. Therefore, DDST can be used as a rapid technique for the preliminary identification of and subsequently appropriate treatment for MDR isolates. While it is hard to foresee the effect of MBL genes in the future of antimicrobial treatment, there is uncertainty that the mainstay antibiotic regimen used to eradicate *P*. *aeruginosa* infections can be depended on to control infections by this organism. MBL strains have been found to be correlated with a rapid onset of infection and a rapid trajectory towards mortality, [16] an indication of their importance. Moreover, there is the added risk of transfer of MBL genes from *P. aeruginosa* to other Enterobacteriaceae. While non-MBL-producing carbapenem-resistant *P. aeruginosa* is not common, such isolates were identified in 22.7% of the isolates in this study. 

**Figure 1:**
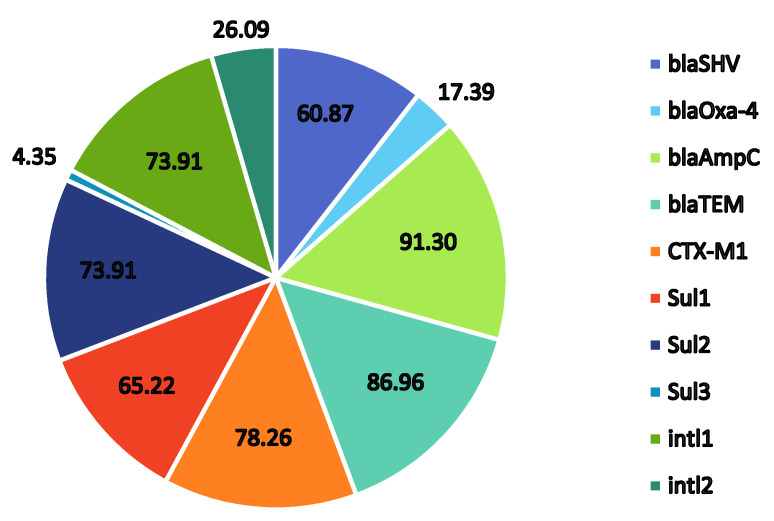
Graphical representation of the % distribution frequencies of antibiotic resistance genes detected in twenty-three *Pseudomonas* sp. (22 *P. aeruginosa* and 1 *P. putida*) isolated from Sodwana Bay, South Africa.

The treatment of *P*. *aeruginosa* is becoming increasingly complex, particularly in hospital situations, because of the inherent and developed resistance to most treatments. In the present study, the *bla_AmpC_* genes were found in 20 (86.9%), *bla_TEM_* in 19 (82.6%), *bla_Oxa_*_-4_ in 4 (18.2%), *CTX-M1* in 17 (73.9%), and *bla_SHV_* in 14 (64%) *P. aeruginosa* tested, confirming ESBL production (Figure 1). The single *P. putida* isolate (OSW2) was found to carry *bla_AmpC_*, *bla_TEM_*, and *CTX-M1* genes. All the isolates from this study carried at least two resistance genes responsible for ESBL production (Table 2). Shakibaie et al. reported that 6.6% and 2.5% of ESBL-producing *P. aeruginosa* isolates carried *bla_SHV_*, and *bla_TEM_* family genes, respectively [24]. CTX-M family members are also known to hydrolyze cefotaxime and ceftriaxone [25]. In South America, Europe, as well as Asia, the CTX-M 1 is most frequently reported [25,26] in *E. coli* isolates. Notably, the majority of the *P. aeruginosa* isolates in the study were ESBL strains with the co-production of AmpC of which only one from a diver with OE (OE6) did not carry the *bla_Amp_* gene. Cephamycins including cefoxitin and cefotetan, oxyimino–cephalosporins including ceftazidime, cefotaxime, and ceftriaxone, and monobactams such as aztreonam are hydrolyzed by AmpC β-lactamases [27]. Therefore, the coexistence of ESBLs and AmpC has significant implications in the treatment of these isolates. While molecular methods such as PCR are better suited to the detection of such strains, it is suggested that divers can easily be screened using DDST incorporating 4th generation cephalosporins. 

### 2.4. Presence of Trimethoprim–Sulfonamide-Resistant Genes

Sulfonamide-resistant genes were found at varied frequencies with *sul1* at 63.6%, *sul2* at 77.3%, and *sul3* at 4.6% as seen in Figure 1. These findings suggest cause for concern as although all the isolates were susceptible to Bactrim, the presence of the sulfonamide-resistant genes in *P*. *aeruginosa* isolates could result in their transfer within and between other bacterial species present in the same environmental niche. It appears that the most prevalent *sul* gene in this study was *sul2* followed closely by *sul1.* The resistant gene *sul3* was only found in a single isolate PW1. These results correlate with the findings by Suzuki et al. [28] in which *sul1* and *sul2* were found to be the major genes in total (culturable and non-culturable) bacterial assemblages from aquatic environments in the Durban area of KwaZulu Natal, South Africa. Moreover, their study also reported a low abundance of *sul3* in total bacterial assemblages at most sites, which was in contrast to previous studies in the Philippines indicating a high prevalence of *sul3* in isolates from seawater.

Interestingly, many of the isolates sharing the same ecological niche showed different resistance patterns such as PW1, PW2, and PW3. Such diversity in the gene pool is likely responsible for the MDRce in these isolates facilitated through horizontal gene transfer (HGT) events. Integrons are genes linked to transposons, plasmids, and chromosomes. 

They are responsible in particular for generating antibiotic resistance in pathogenic Gram-negative bacterial infections. *Int1l* in our study was observed in 17 (77.3%) of the isolates while *Intl2* appeared in 6 (27.3%) of the isolates. We observed four (18.2%) isolates with both *Intl1* and *Intl2.* Xu et al. [29] were able to detect 2.54% of *P. aeruginosa* that contained both *Intl1* and *Intl2* in hospital patients in China. Only three isolates, BPT2, OBW 1, and OBW3 did not carry either *Intl1* or *Intl2* genes suggesting the prominent role integrons play in MDRce at Sodwana Bay. Integrons hold a drug resistance gene cassette for various drug groups, and class 1 integrons play a prominent role as a source of resistance genes in both Gram-negative and Gram-positive species. It was therefore not surprising to observe a high abundance of the *Intl1* gene in the *P. aeruginosa* isolates from this study. Of these isolates, 11 (50%) also carried the *sul1* gene, which is the most prominent integron in clinical isolates. Irrespective of whether the isolates carried class 1, class 2, or both classes of integrons, all, except isolate PW3, had at least one *sul* gene present, highlighting the potential for sulfonamide resistance in the future.

### 2.5. Correlation between Antibiotic Susceptibility and Resistance Genes

**Figure 2:**
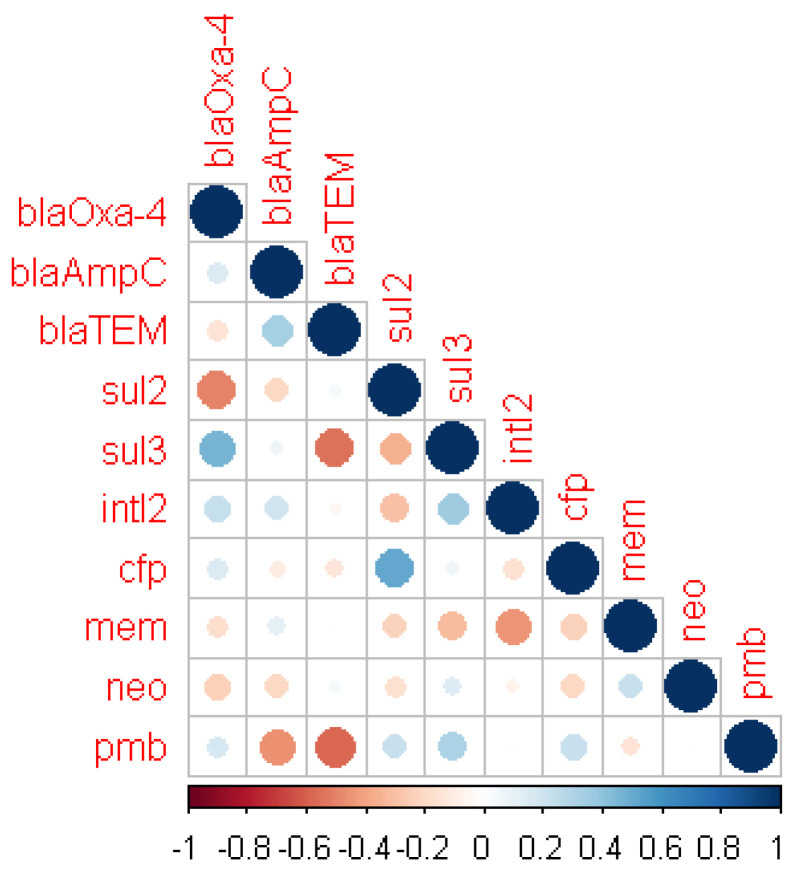
Correlation matrix of antibiotic resistance phenotype and genotypic (antibiotic resistance genes) features showing significant (*p* < 0.05) correlations. Blue circles indicated significant positive correlation and red show significant negative correlation. The size and strength of color represent the numerical value of the correlation coefficient.

Few significant relationships were found between the antibiotic resistance phenotypes and the genotypes (presence of antibiotic resistance genes), including that of β-lactam resistance, despite the high number of resistant isolates seen phenotypically (Table 1). A correlation matrix analysis (Figure 2) of the significant phenotypic antibiotic resistance and genotypes showed few and weak positive relationships between the presence of β-lactamase genes and resistance to β-lactams. Only *bla_Oxa-4_* and *bla_AmpC_* were found to have a weak positive relationship with cefaperazone and meropenem, respectively. The presence of *bla_TEM_* had a predominantly negative relationship with all other genes except for *bla_AmpC_* where it showed a strong positive relationship. Sulfonamide resistance gene, *sul3,* exhibited a strong positive relationship with *bla_Oxa-4_*, while *sul2* exhibited an equally strong negative relationship with the β-lactamase gene. The positive relationship of *intl2* with *sul3*, *bla_Oxa-4_*, and *bla_AmpC_* further provides evidence for the prominent role played by integrons in MDRce.

Resistance to meropenem has a negative association with both polymyxin B and cefaperazone, while meropenem and neomycin share a positive relationship. Other significant positive correlations between polymyxin B and cefaperazone also indicate the co-occurrence of resistance, which contributes to MDRce in *P. aeruginosa* in Sodwana Bay.

An exhaustive profiling of resistance genotypes to different classes of antibiotics is necessary to determine the extent of MDRce in *P. aeruginosa*. Amplification of, for example, *aac(3)-III* (aminoglycoside resistance), *qnrA*, *qnrB*, *qnrS* (quinolone resistance), *tet* (tetracycline resistance), and *blaIMP* and *blaVIM* could provide more insight into the antibiotic resistance profiles of the isolates. Additionally, antibiotic resistance due to mutations, rather than the acquisition of antibiotic resistance genes, could be at play here. While the sample size is a limiting factor, this study provides the first evidence of MDR *P. aeruginosa* in Sodwana Bay that results in OE infections in SCUBA divers.

## 3. Materials and Methods

### 3.1. Ethics Statement

The study was conducted in accordance with the Declaration of Helsinki and the study protocol was approved by the Ethics Committee of the University of Johannesburg (Reference number 2018–08-06). All participants gave their informed consent for inclusion in the study.

### 3.2. Sample Collection 

A total of 137 random samples were collected at Sodwana Bay. These included 22 ear swabs from apprentice divers before pool training (BPT); 19 ear swabs after they completed their pool training (APT); 6 ear swabs from apprentice divers who completed some ocean dives (ADO); and 10 ear swabs from apprentice and professional divers who presented with OE (OE) including a visiting tourist with OE (T_w_OE). In addition, 10 samples of water were collected from the training pool (PW) and 70 samples of seawater were collected from different surface distances in the vicinity of the dive (OSW), 30 of which were collected at depths between 15 and 22 m (OBW). The water samples were collected in 1000 mL sterile pressure resistant Schott bottles. The samples were then placed on ice and transported to the laboratory for further analysis within 24 h.

### 3.3. Isolation and Identification of P. aeruginosa

The ear swabs were incubated in nutrient broth (NB) at 37 °C for 24 h. After incubation the cultures were streaked onto sterile nutrient agar (NA) plates and *Pseudomonas aeruginosa* cetrimide agar (PACA) followed by overnight incubation at 37 °C for 24–48 h to obtain pure cultures [30]. The water samples were membrane filtered and the impregnated filter paper discs were aseptically placed onto NA and PACA plates followed by incubation at 37 °C for 24–48 h. Bacterial colonies exhibiting a greenish pigmentation (indicative of *P. aeruginosa* pyocyanin production) were morphologically confirmed to be Gram-negative bacilli, and further characterized in duplicate using standard biochemical tests: oxidase, catalase, nitrate reduction, indole, methyl red, Voges–Proskauer, citrate utilization, and glucose fermentation tests [15,16]. 

The isolates were also identified by 16S rDNA sequencing using the universal forward primer 16S-27F-AGAGTTTGATCMTGGCTCAG and reverse primer 16S-1492R- CGGTTACCTTGTTACGACTT. Fragments were analysed on an ABI 3500xl Genetic Analyzer (Applied Biosystems, ThermoFisher Scientific) and 16S rDNA sequences aligned to reference bacterial species using Basic Local Alignment Search Tool (BLAST) available at http://www.ncbinlm.nih.gov/ (accessed date 23 December 2021) for identification. 

### 3.4. Antimicrobial Sensitivity Testing

Antimicrobial susceptibility tests were performed by the disk diffusion method for the 22 isolates confirmed to be *P. aeruginosa* using commercially available antibiotic disks (Manchester, UK) according to the Clinical Laboratory Standard Institute guidelines (2018) (CLSI, 2018). The following antibiotics, which included those generally prescribed by medical practitioners in Sodwana Bay to treat patients with ear infections (possibly OE), were used: β-lactams: Cefotaxime 30 μg, Cefaperazone 30 µg, Ceftazidime 30 μg, Meropenem 10 μg, Amoxicillin 30 µg, Augmentin (Amoxicillin–Clavulanic acid) 30 µg, Aztreonam 30 µg, Imipenem 10 µg, Piperacillin–Tazobactam 36 μg, Penicillin G 10 U; Non *β*-lactams: Neomycin 30 µg, Ciprofloxacin 10 µg, Amikacin 30 µg, Polymyxin B 300 U, Ofloxacin 5 µg, and Bactrim (trimethoprim–sulfamethoxazole (1.25/23.75)). *P. aeruginosa* ATCC 27853 was used as the positive control. Mueller–Hinton agar plates were incubated at 37 ℃ for 18 h, after which they were observed for the presence of zones of growth inhibition around the different antibiotic disks [31]. The results were interpreted using the recommendations from the guidelines M26-A and M-100S 27 of the Clinical and Laboratory Standard Institute [31]. Zone diameter breakpoints for *P. aeruginosa* within the different classes of antibiotics used in this study can be found in Supplementary Table S1.

### 3.5. Phenotypic Detection of ESBLs and MBLs

The Double Disk Synergy Test (DDST) was used to detect MBL production in all isolates confirmed to be *P. aeruginosa* and unaffected by imipenem and/or meropenem. For MBL production, disks comprising imipenem-EDTA were placed on lawn cultures grown on Mueller–Hinton agar plates as recommended by the CLSI. MBL-positive isolates were defined as those with a difference of ≥7 mm in diameter between imipenem-EDTA and imipenem-only disk inhibition [32]. The EDTA disc alone, and strains of *P. aeruginosa* that are positive for VIM-1 MBL production, were used as controls.

### 3.6. PCR Detection of Genes Associated with Antimicrobial Resistance 

The prevalence of β-lactamase genes TEM, SHV, OXA, CTX-M, and AmpC was determined by PCR amplification following the method of Abrar et al. (2019) [33]. Genes, *sul1*, *sul2,* and sul3 associated with trimethoprim–sulfonamide resistance were examined in isolates according to Jouini et al. [34]. The presence of *IntI1* and *IntI2* genes encoding classes 1 and 2 integrases, respectively, were identified by PCR according to Machado et al. [35]. Positive and negative controls were kindly provided by the University of Johannesburg, Doornfontein, Johannesburg, and were used in all PCR experiments.

### 3.7. Statistical Analysis

Antibiotic susceptibility and resistance gene profiles were converted into a binary coding system for the purpose of statistical analysis. Resistance to an antibiotic was represented as one, while susceptibility was assigned a zero. Similarly, the presence of a specific antibiotic resistance gene was assigned a one, while absence of the gene was assigned a zero. Correlations between the phenotypes and genotypes were calculated using the ‘cor’ function and the significance (Pearson method) determined using the ‘corr.test’ function in the open statistical program R (v4.1.2) [36]. Correlations of significant relationships (*p*-value of <0.05 was considered statistically significant) were further visualized using the ‘corrplot’ function from the ‘corrplot’ R package (v0.92) [37]. 

## 4. Conclusions

In our research, the increased rate of ESBLs found in *P*. *aeruginosa* isolated from SCUBA divers, swimming pool water, and ocean water in Sodwana Bay, South Africa demonstrated a rise in MDRce, which subsequently impacts the treatment of OE using popular pharmaceuticals such as imipenem. Although all the isolates were found to be susceptible to Bactrim (trimethoprim–sulfamethoxazole), the high prevalence of *sul* genes and class 1 and 2 integrons suggests the potential for bacteria in these environmental niches to develop resistance to sulfonamides in the future. This will lead to enhanced spread of infection, complexities of clinical conditions, and therapy failures in our institutions.

## Figures and Tables

**Table 1:** Antimicrobial resistance of *Pseudomonas aeruginosa* isolates from divers, pool, and ocean water to different classes of antibiotics.

Antibiotics	Abbreviation	Total Number of Resistant Isolates n = 22	
		S No. (%)	R No. (%)
*β-lactams*			
Cefotaxime	*cfx*	0(0)	22(100)
Cefaperazone	*cfp*	2(9.1)	20(90.9)
Ceftazidime	*caz*	6(227.3)	16(72.7)
Meropenem	*mem*	10(45.5)	12(54.5)
Amoxicillin	*amx*	0(0)	22(100)
Augmentin	*amc*	0(0)	22(100)
Aztreonam	*atm*	4(18.2)	18(81.8)
Imipenem	*ipm*	0(0)	22(100)
Piperacillin–Tazobactam	*tzp*	0(0)	22(100)
Penicillin G	*pen*	0(0)	22(100)
*Non β-lactams*			
Neomycin	*neo*	10(45.5)	12(54.5)
Ciprofloxacin	*cip*	0(0)	22(100)
Amikacin	*amk*	0(0)	22(100)
Polymyxin B	*pmb*	15(68.2)	7(31.8)
Ofloxacin	*ofx*	0(0)	22(100)
Bactrim (Trimethoprim–sulfamethoxazole)	*sxt*	22(100)	0(0)

**Table 2:** Presence or absence of resistance genes in *Pseudomonas aeruginosa* isolates.

	Resistant Gene ^1^									
Isolate ^2^	*bla_SHV_*	*bla_Oxa-4_*	*bla_AmpC_*	*bla_TEM_*	*CTX-M1*	*Sul1*	*Sul2*	*Sul3*	*intl1*	*intl2*
**BPT2**	+	-	-	-	+	-	+	-	-	-
**APT1**	+	-	+	+	+	+	+	-	+	-
**APT2**	-	-	+	+	+	+	+	-	-	+
**ADO1**	+	+	+	+	+	+	-	-	+	-
**ADO2**	-	-	+	+	+	+	-	-	+	+
**OE1**	-	-	+	+	+	-	+	-	+	-
**OE2**	-	+	+	+	+	-	-	-	+	-
**OE3**	+	-	+	+	+	-	+	-	-	-
**OE4**	-	-	+	+	+	+	+	-	+	-
**OE5**	+	-	+	+	+	-	+	-	+	-
**OE6**	+	-	-	+	+	+	+	-	+	-
**OE8**	+	-	+	+	-	-	+	-	+	-
**T_w_OE1**	+	-	+	+	+	+	+	-	+	-
**PW1**	+	+	+	-	-	+	-	+	+	+
**PW2**	-	-	+	+	+	+	+	-	+	-
**PW3**	+	-	+	+	+	-	-	-	+	+
**OSW1**	+	+	+	+	-	+	+	-	+	+
**OSW2 ^3^**	-	-	+	+	+	+	+	-	+	-
**OSW3**	+	-	+	+	+	+	+	-	+	-
**OBW1**	+	-	+	+	+	+	-	-	-	-
**OBW2**	+	-	+	-	+	+	+	-	+	-
**OBW3**	-	-	+	+	-	-	+	-	-	-
**OBW4**	-	-	+	+	-	+	+	-	-	+

^1^: + indicates presence; - indicates absence. ^2^: BPT—before pool training; APT—after pool training; ADO—after ocean dive; OE—divers with otitis externa; T_w_OE—tourist diver with otitis externa; PW—pool water; OSW—ocean surface water; OBW—ocean water below 20 m ^3^: OSW2—identified as *P. putida.*

## Data Availability

On request from the principal investigator, the data described in this study will be provided.

## Supplementary Materials

The following supporting information can be downloaded at: https://www.mdpi.com/article/10.3390/pathogens11010091/s1, Table S1: Zone diameter breakpoints to different classes of antibiotics used in this study based on the CLSI guidelines for Pseudomonas aeruginosa [Table 2B-1, 31].

## References

[1] Zhang Y., Wang Q., Low W., Wang Y., Zhu X. (2013). Microbiological safety of household membrane water filter. J. Environ. Biol..

[2] Azizi M.H. (2011). Ear disorders in Scuba Divers. Int. J. Occup. Environ. Med..

[3] Hlavsa C.M., Cikesh L.B., Roberts A.V., Kahler M.A., Vigar M., Hilborn D.E., Wade J.T., Roellig D.M., Murphy L.J., Xiao L. (2018). Outbreaks associated with treated recreational water—United States, 2000–2014. Morb. Mortal. Wkly. Rep..

[4] D’Agata E., Mandell E.J., Dolin R., Blaser M.J. (2017). Pseudomonas aeruginosa and other pseudomonas species. Infectious Disease Essentials.

[5] Geyer M., Howell-Jones R., Cunningham R., McNulty C. (2011). Consensus of microbiology reporting of ear swab results to primary care clinicians in patients with otitis externa. Br. J. Biomed. Sci..

[6] Rossolini G.M., Mantengoli E. (2005). Treatment and control of severe infections caused by multi-resistant *Pseudomonas aeruginosa*. Clin. Microbiol. Infect..

[7] Pillay D., Jardine N.P. (2010). Recreational scuba divers knowledge regarding the audiological consequences of the sport. S. Afr. J. Sports Med..

[8] Kumar S.P., Singh U. (2015). Malignant otitis externa: A review. J. Infect. Dis. Ther..

[9] Illing E., Olaleye O. (2011). Malignant otitis externa: A review of aetiology, presentation, investigations and current management strategies. Webmed Cent. Otorhinolaryngol..

[10] Zowalaty E.E.M., Thani A.A.A., Webster J.T., Zowalaty E.E.A., Schweizer P.H., Nasrallah N.G., Marei H.E., Ashour M.H. (2015). *Pseudomonas aeruginosa*: Arsenal of resistance mechanisms, decades of changing resistance profiles, and future antimicrobial therapies. Future Microbiol..

[11] Puzniak L., De Pestel D.D., Srinivasan A., Ye G., Murray J., Merchant S., De Ryke C.A., Gupta V. (2019). A combination antibiogram evaluation for *Pseudomonas aeruginosa* in respiratory and blood sources from intensive care unit (ICU) and non-ICU settings in U.S. hospitals. Antimicrob. Agents Chemother..

[12] Tacconelli E., Carrara E., Savoldi A., Harbarth S., Mendelson M., Monnet D.L., Pulcini C., Kahlmeter G., Kluytmans J., Carmeli Y. (2018). WHO Pathogens Priority List Working Group. Discovery, research, and development of new antibiotics: The WHO priority list of antibiotic-resistant bacteria and tuberculosis. Lancet Infect. Dis..

[13] Kali A., Srirangaraj S., Kumar S., Divya H.A., Kalyani A., Umadevi S. (2013). Detection of metallo-*β*-lactamase producing *Pseudomonas aeruginosa* in intensive care units. Aust. Med. J..

[14] Barbier F., Andremont A., Wolff M., Bouadma L. (2013). Hospital-acquired pneumonia and ventilator-associated pneumonia: Recent advances in epidemiology and management. Curr. Opin. Pulm. Med..

[15] Pachori P., Gothalwal R., Gandhi P. (2019). Emergence of antibiotic resistance *Pseudomonas aeruginosa* in intensive care unit; a critical review. Genes Dis..

[16] Shu J.-C., Kuo A.-J., Su L.-H., Liu T.-P., Lee M.-H., Su I.-N., Wu T.-L. (2017). Development of carbapenem resistance in *Pseudomonas aeruginosa* is associated with *OprD* polymorphisms, particularly the amino acid substitution at codon 170. Antimicrob. Chemother..

[17] Adjei C.B., Govinden U., Moodley K., Essack S.Y. (2018). Molecular characterisation of multidrug-resistant *Pseudomonas aeruginosa* from a private hospital in Durban, South Africa. S. Afr. J. Infect. Dis..

[18] Ekwanzala M.D., Dewar J.B., Kamika I., Momba M.N.B. (2018). Systematic review in South Africa reveals antibiotic resistance genes shared between clinical and environmental settings. Infect Drug Resist..

[19] Kao C.-Y., Chen S.-S., Hung K.-H.H., Wu H.-M., Hsueh P.-R., Jing-Jou Yan J.-J., Wu J.-J. (2016). Overproduction of active efflux pump and variations of OprD dominate in imipenem resistant *Pseudomonas aeruginosa* isolated from patients with bloodstream infections in Taiwan. BMC Microbiol..

[20] Tabar M.M., Mirkalantari S., Amoli R.I. (2016). Detection of ctx-M gene in ESBL producing *E. coli* strains isolated from urinary tract infection in Semnan, Iran. Electron. Physician.

[21] Varriale L., Dipineto L., Russo T.P., Borrelli L., Romano V., D’Orazio S., Pace A., Menna L.F., Fioretti A., Santaniello A. (2020). Antimicrobial resistance of *Escherichia coli* and *Pseudomonas aeruginosa* from companion birds. Antibiotics.

[22] Meng L., Liu H., Lan T., Dong L., Hu H., Zhao S., Zhang Y., Zheng N., Wang J. (2020). Antibiotic resistance patterns of *Pseudomonas* spp. isolated from raw milk revealed by whole genome sequencing. Front. Microbiol..

[23] Qin X., Zerr D.M., McNutt M.A., Berry J.E., Burns J.L., Kapur R.P. (2012). *Pseudomonas aeruginosa* syntrophy in chronically colonized airways of cystic fibrosis patients. Antimicrob. Agents Chemother..

[24] Shakibaie M.S.F., Hashemi A., Adeli S. (2008). Detection of TEM, SHV and PER type extended-spectrum beta-lactamase genes among clinical strains of *Pseudomonas aeroginosa* isolated from burnt patients Shafa hospital, Kerman, Iran. Iran J. Basic Med. Sci..

[25] Rossolini G.M., D’Andrea M.M., Mugnaioli C. (2008). The spread of CTXM-type extended-spectrum beta-lactamases. Clin. Microbiol. Infect..

[26] Dabir S., Mohammadi J., Alizadeh A., Karimi F., Nori K., Mahmoudi S., Pournajafi A. (2020). Investigation of the prevalence of CTX-M-1 beta-lactamase gene in *Pseudomonas aeruginosa* strains isolated from urinary tract infections in Zanjan Hospitals, Iran. S. Asian Res. J. Biol. Appl. Biosci..

[27] Hsieh W.-S., Wang N.-Y., Feng J.-A., Weng L.-C., Wu H.-H. (2015). Identification of DHA-23, a novel plasmid-mediated and inducible AmpC beta-lactamase from Enterobacteriaceae in Northern Taiwan. Front. Microbiol..

[28] Suzuki S., Ogo M., Koike T., Takada H., Newman B. (2015). Sulfonamide and tetracycline resistance genes in total and culturable bacterial assemblages in South African aquatic environments. Front. Microbiol..

[29] Xu Z., Li L., Shirtliff M.E., Alam M.J., Yamasaki S., Shi L. (2009). Occurrence and characteristics of class 1 and 2 integrons in *Pseudomonas aeruginosa* isolates from patients in Southern China. J. Clin. Microbiol..

[30] Quinn P.J., Markey B.K., Leonard F.C., Fitz Patrick E.S., Fanning S., Hartigan P.J. (2011). Veterinary Microbiology and Microbial Diseases.

[31] Clinical and Laboratory Standards Institute (2017). Methods for Determining Bactericidal Activity of Antimicrobial Agents Approved Guideline.

[32] De S.A., Kumar H.S., Baveja M.S. (2010). Prevalence of metallo-*β*-lactamase producing *Pseudomonas aeruginosa* and *Acinetobacter* species in intensive care areas in a tertiary care hospital. Indian J. Crit. Care Med..

[33] Abrar S., Ain A.U., Liaqat H., Hussain S., Rasheed F., Riaz S. (2019). Distribution of blaCTX—M, blaTEM, blaSHV and blaOXA genes in extended-spectrum-β-lactamase-producing clinical isolates: A three-year multi-center study from Lahore, Pakistan. Antimicrob. Resist. Infect. Control.

[34] Jouini A., Vinué L., Slama K.B., Sáenz Y., Klibi N., Hammami S., Boudabous A., Torres C. (2007). Characterization of CTX-M and SHV extended-spectrum beta-lactamases and associated resistance genes in *Escherichia coli* strains of food samples in Tunisia. J. Antimicrob. Chemother..

[35] Machado E., Cantón R., Baquero F., Galán J.C., Rollán A., Peixe L., Coque T.M. (2005). Integron content of extended-spectrum-beta-lactamase-producing *Escherichia coli* strains over 12 years in a single hospital in Madrid, Spain. Antimicrob. Agents Chemother..

[36] R Foundation for Statistical Computing, R Core Team (2021). R: A Language and Environment for Statistical Computing.

[37] Wei T., Simko V. (2021). R Package ‘Corrplot’: Visualization of a Correlation Matrix.

