# Supplementary material for: Antimicrobial Susceptibility Profiles among Pseudomonas aeruginosa Isolated from Professional SCUBA Divers with Otitis Externa, Swimming Pools and the Ocean at a Diving Operation in South Africa
Source: Pathogens. 2022 Jan 13;11(1):91. doi: 10.3390/pathogens11010091 (PMC8777857; doi:10.3390/pathogens11010091)
Supplement: Supplementary file 1 [file pathogens-11-00091-s001.zip › pathogens-1499632-supplementary.pdf]

Table S1. Zone diameter breakpoints to different classes of antibiotics used in this study based on the CLSI guidelines for *Pseudomonas aeruginosa* [Table 2B-1, 31].

Antimicrobial class	Mode of action	Antimicrobial agent	Interpretive criteria and zone diameter breakpoints to the nearest mm		
			S	I	R
Penicillins	Inhibit cell wall synthesis	Penicillin G	-	-	-
β-lactam	Inhibit cell wall synthesis	Amoxicillin	-	-	-
β-lactam combination agent	Inhibit cell wall synthesis	Augmentin	-	-	-
		Piperacillin-tazobactam	≥21	15–20	≤14
Cephems	Inhibit cell wall synthesis	Cefotaxime	-	-	-
		Cefaperazone	-	-	-
		Ceftazidime	≥18	15–17	≤14
Carbapenem	Inhibit cell wall synthesis	Meropenem	≥19	16–18	≤15
		Imipenem	≥19	16–18	≤15
Monobactam	Inhibit cell wall synthesis	Aztreonam	≥22	16–21	≤15
Aminoglycosides	Inhibit protein synthesis	Amikacin	≥17	15–16	≤14
		Neomycin	-	-	-
Fluoroquinolones	Inhibit bacterial DNA synthesis and replication	Ciprofloxacin	≥25	19–24	≤18
		Ofloxacin	≥16	13–15	≤12
Lipopeptides	Disruption of cell wall integrity	Polymyxin B	-	-	-
Folate pathway antagonists	Inhibit folate action	Trimethoprim-sulfamethoxazole	-	-	-

S: Susceptible, I: Intermediate, R: Resistance; -: zone diameter breakpoints not available, results were interpreted based on zone diameter breakpoints for antimicrobial agents belonging to the same class.
